# Umbilical cord blood-derived CD11c^+^ dendritic cells could serve as an alternative allogeneic source of dendritic cells for cancer immunotherapy

**DOI:** 10.1186/s13287-015-0160-8

**Published:** 2015-09-25

**Authors:** Jeetendra Kumar, Vaijayanti Kale, Lalita Limaye

**Affiliations:** Stem Cell Laboratory, National Centre for Cell Science, Ganeshkhind, Pune, 411007 India

## Abstract

**Introduction:**

Allogenic dendritic cells (DCs) generated from healthy donors, who are complete or partially HLA-matched, have been used for clinical trials. One of the sources for allogenic DCs is umbilical cord blood (UCB) cells. However, as far as cord blood cells are concerned, looking at their naïve nature, there is a concern as to whether the DCs generated from them will have enough potential to elicit a proper T cell response. For this, we compared CD11c^+^ UCB-DCs/ Cytotoxic T lymphocytes (CTLs) with the conventional source, i.e. peripheral blood (PBL) monocyte DCs/CTLs, using various parameters.

**Methods:**

CD11c^+^ DCs generated from the two sources were compared morphologically, phenotypically and functionally. Functional assays included antigen uptake, chemotactic migration and MLR (mixed lymphocyte reaction). The CTLs generated were examined for the activation markers, granzyme A & granzyme B, and IFN-γ secretion. MUC1 (STAPPVHNV) peptide-specific CTLs were quantified by Streptamer staining. *In vitro* CTL activity was assessed by their efficiency in killing MCF-7 cells. For *in vivo* CTL assay, a xenograft of MCF-7-luc-F5 cells in female NOD/SCID mice was employed. Regression of tumors in mice was monitored using an in vivo imaging system before and after ten days of CTL infusion. Statistical analysis of all the experiments between the two groups was evaluated by one-way ANOVA.

**Results:**

The CD11c^+^ DCs from the two sources were morphologically and phenotypically similar. Their capacity to uptake antigen, migration towards CCL-19 and MLR activity were equivalent. UCB-CTLs had significantly higher levels of activation markers, number of MUC1 specific CTLs, IFN-γ secretion and IL-12p70/IL-10 ratio than that of PBL-CTLs. Hematoxylin and Eosin-stained tumor sections showed T cell infiltration, which was further confirmed by immunofluorescence staining. *In vivo* CTL activity was found to be similar with the two sources.

**Conclusions:**

Our data demonstrate that CD11c^+^ UCB-DCs/CTLs are as potent as standard CD11c^+^ PBL-DC/CTLs and could therefore be used as an allogenic source for therapeutic purposes. The findings of this study could help in taking us one step closer towards the personalized therapy using DC based cancer vaccines.

**Electronic supplementary material:**

The online version of this article (doi:10.1186/s13287-015-0160-8) contains supplementary material, which is available to authorized users.

## Introduction

Dendritic cells (DCs) are crucial for the induction of both primary and secondary immune responses, as well as for eliciting immunological tolerance. Their capacity to effectively cross-present exogenous antigens to T lymphocytes makes DCs essential for the induction of adaptive immune responses against malignant cells. This unique attribute of DCs has offered the possibility of developing clinical protocols involving DCs for use in cancer immunotherapy.

DCs were introduced as adjuvants in vaccination strategies that aimed to induce antigen-specific effector and memory cells. DC therapy represents a new and promising immunotherapeutic approach for the treatment of advanced cancers. In the last two decades, large numbers of clinical trials have been conducted using DC vaccines targeting different kinds of tumors, and it was found that they were able to initiate promising clinical responses against a number of diseases, like renal cell carcinoma, melanoma, HIV, multiple myeloma, acute myeloid leukemia, breast cancer etc. [1–13]. Immunotherapies with allogeneic DCs pulsed with tumor antigens to generate specific T cell responses have been tested in clinical trials with patients having solid tumors as well as in different hematological malignancies [14, 15]. Allogeneic DCs can be generated from CD34^+^ cells derived from umbilical cord blood (UCB) [16–28]. Thus, UCB could be exploited as an additional source for the generation of allogeneic DCs. UCB-derived DCs have been used in the pilot phase of clinical trials as well, in hematological disorders like AML, as a therapeutic agent to increase the survival of patients [29, 30].

We have earlier standardized methods for the large scale generation of DCs from UCB-derived CD34^+^ cells and mononuclear cells (MNCs), [25, 26] and DCs with enhanced functionality [31]. These DCs were characterized by immunophenotyping and functional assays like mixed lymphocyte reaction (MLR), antigen uptake and chemotactic migration. However, for efficacious DC vaccines, the basic requirement is that the DCs should generate effector and memory cytotoxic T lymphocytes (CTLs), to elicit a comprehensive immune response. The standard treatment procedures utilize peripheral blood (PBL) monocyte-derived DCs. There are very few reports where the potency of UCB-derived DCs has been compared with PBL monocyte-derived DCs [32, 33]. Therefore, here we report a systematic study of a comparison between UCB-DCs/CTLs and PBL-DCs/CTLs, using various parameters. As the basis of CTL assay is HLA-A*0201-restricted, which is a major histocompatibility complex (MHC) class I polymorphism, we generated DCs from HLA-A*0201positive PBL/UCB samples. We then carried out in-depth characterization and functionality tests with these DCs/CTLs. Data generated from this study clearly demonstrate that UCB-DCs are as potent as their standard vaccine counterparts i.e., PBL monocyte DCs, and could therefore be used as an allogenic source for therapeutic applications.

## Materials and methods

### Materials

#### Cytokines

The recombinant human cytokines used for the study were fms-like tyrosine kinase-3 ligand (Flt-3L), thrombopoietin (TPO), stem cell factor (SCF), IL-4, IL-2, IL-7, granulocyte-macrophage colony stimulating factor (GM-CSF), TNF-α, CD40L (CD40 ligand) and macrophage inflammatory protein 3β (CCL-19). All recombinant human cytokines were purchased from Peprotech Asia, Rehovot Israel.

#### Antibodies

The antibodies used for flow cytometry were mouse anti-human mAbs: CD1a, CD25, CD11c, CD40, CD8 - antigen presenting cell (APC)-tagged; CD58, CD54, CD80, CD83, CD86, HLA-A2, CD45RA, granzyme A - phycoerythrin (PE)-tagged; CD3, HLA-DR, HLA-ABC, granzyme B - fluorescein isothiocyanate (FITC)-tagged and CD 69 PECy7-tagged. For immunofluorescence staining primary antibody was mouse anti-human purified CD3 and secondary antibody was goat anti-mouse tagged with Alexa Fluor® 488 (Life technologies, MA, USA). All monoclonal antibodies and respective isotype controls used for the study were purchased from BD pharmingen (San Diego, CA, USA).

#### Cell lines

MCF-7 was used in the cell lysate preparation, *in vitro* and *in vivo* CTL assay. MCF-7-luc-F5 (Caliper Life Sciences, Hopkinton, MA, USA) was used for *in vivo* CTL assay using *in vivo* imaging system (IVIS). H1229 and SH-SY5Y were used as nonspecific targets for the CTLs generated from the MCF-7 lysate-pulsed DCs.

#### Culture medium and other reagents used

Iscove’s modified Dulbecco’s medium (IMDM), Roswell Park Memorial Institute-1640 (RPMI-1640), (DMEM), Dulbecco's modified Eagle's medium/nutrient F-12 Ham 1:1 nutrient mixture (DMEM/F-12 media) and IMDM without phenol red were procured from Sigma Aldrich, St. Louis, MO, USA. Histopaque (ρ-1.007g/ml), FITC-labeled dextran (40 kDa), Wright Stain, Giemsa Stain, Hematoxylin Solution-Mayer’s, Eosin Y solution, DPX mountant, lipopolysaccharide (LPS), keyhole limpet hemocyanin (KLH), dimethyl sulfoxide (DMSO), PPO, POPOP were all from Sigma Aldrich, St. Louis, MO, USA; ELISA kit (BD OptEIA); heparin (SRL Pvt. Limited Mumbai, India); Rosette Sep for T cell isolation (Stem Cell Technologies, Vancouver, Canada); [^3^H] thymidine (240 GBq/ milli mole, BRIT, Navi Mumbai, India); Intracellular fixation & permeabilization buffer set (ebiosciences San Diego, CA, USA) Steptramers (Iba-lifesciences, Goettingen, Germany), Luciferin D Potassium Salt (Perkinelmer, Waltham, MA, USA), reduced growth factor Matrigel (Becton-Dickinson), HBSS buffer (Life technologies, MA, USA) and CRYO-OCT compound (Thermo Fisher Scientific Inc. MA, USA).

## Methods

### Ethical statements for use of human samples

Protocols for collection and processing of all human samples used in this study were approved by the Institutional Ethics Committee (IEC of NCCS) and Institutional Committee for Stem Cell Research (IC-SCR of NCCS). The guidelines followed by these committees are in accordance with the Declaration of Helsinki. Prior informed written consent was obtained from the donors. The human samples used in this study were as follows:

1. Umbilical cord blood: these were used for generating DCs and for isolating autologous T cells for CTL assay
2. Peripheral blood: these samples were used to isolate MNCs and T cells for use in MLR assays
3. Buffy coat: these were used for generating DCs and for isolating autologous T cells for CTL assay

### Collection of blood samples

Collection of blood samples was as follows:

1. Umbilical cord blood (UCB): the samples were collected in preservative-free heparin (40 IU of heparin/ ml of blood) with plain IMDM in sterile containers. Then samples were brought to the laboratory on ice packs and were processed to isolate MNCs
2. Peripheral blood (PBL): buffy coat bags were brought to the laboratory on ice packs and were subsequently processed to isolate MNCs

### Processing of blood samples

Processing of blood samples was as follows:

1. Isolation of MNC from UCB and PBL: UCB MNCs and PBL MNCs were separated by Histopaque® density gradient centrifugation. Turk’s solution was used to take nucleated cell counts, using a hemocytometer. MNCs thus obtained were used for DC generation
2. Isolation of T-cells from peripheral blood: T cells were isolated from blood for MLR assay, using the RosetteSep™ (negative selection kit), according to the manufacturer’s instructions

#### In vitro *generation and culture of DCs from UCB*

DCs were generated from umbilical cord blood samples by our method as described earlier [25, 26, 31]. Briefly, MNCs from peripheral blood samples were seeded at a density of 10^7^ cells/2 ml/well in six-well plates. Monocytes were enriched by plastic adherence and were used for DC generation. The terminal differentiation of precursor cell populations to DCs were induced by subsequently culturing them with GM-CSF (50 ng/ml) and IL-4 (30 ng/ml) for 3 days and GM-CSF (50 ng/ml) + TNF-α (50 ng/ml) for 4 days in IMDM supplemented with 5 % autologous/AB+ plasma. Immature DCs were harvested on day 5 and were used for antigen uptake assay. On day 7, the cells were subjected to maturation with a combination of pro-inflammatory signal, a combination of TNF-α, LPS and CD40L, at the concentration of 100 ng /ml each, for 48 h. Mature DCs were used for all other assays. Experimental design for generation of DCs is given in a flow chart in Additional file 1: Figure S1.

### *In vitro* generation and culture of DCs from PBL

Mononuclear cells were obtained by histopaque density gradient centrifugation of buffy coat samples. MNCs were seeded at the density of 10^7^ cells/well in six-well plates containing 2 ml of IMDM supplemented with 1 % human AB+ plasma. The cells were kept for 1.5 h at 37 °C in a 5 % CO2 incubator and the non-adherent and the loosely adherent cells were removed by washing with IMDM. The adherent cells were used for DC generation in a similar way as described for the UCB samples. The experimental design for generation of DCs is given in a flow chart in Additional file 1: Figure S1.

#### Morphological and phenotypic analysis

Morphological characterization of DCs was done by staining with Wright and Giemsa stains and observing under a microscope. For phenotypic analysis, the cells were stained with a panel of ten antibodies namely CD1a, CD11c, CD40, CD58, CD54, CD80, CD83, CD86, HLA-DR and HLA-ABC along with appropriate isotype controls and acquired using the FACS Canto II (BD San Jose, CA, USA) [25, 26, 31].

#### Flow Cytometry

For all flow cytometry analysis, the cells were suspended in 100 μL of cold PBS containing 1% BSA. Specific labeled mAbs and appropriate isotype controls were added and the cells were incubated on ice for 30 minutes. Cells were washed thrice with ice cold PBS and re-suspended in 1 % paraformaldehyde. Cells were acquired using the FACS Canto II. Data were analyzed by FACS Diva (BD) and histogram overlays were prepared using FlowJo (LLC, Ashland OR, USA).

#### Functional characterization of DCs

The assays used to access the functionality of *in vitro* generated UCB-DCs and PBL-DCs were as follows:

1. Endocytosis assay with FITC-tagged dextran: we tested the receptor-mediated endocytosis in the generated DCs using FITC-tagged dextran. Immature DCs were harvested on day 5 and incubated with FITC-dextran (20 μg/ml), either at +4 °C (internalization control) or at +37 °C, for 30 and 60 minutes. The cells were then acquired using the FACS Canto II [25, 26, 31]
2. Chemotaxis: the chemotaxis of the *in vitro* generated UCB and PBL-DCs toward CCL-19 was assessed in a 24-well cell culture plate with BD Falcon ™ Cell culture inserts (pore size 8.0 μm) as described earlier [25, 26, 31]
3. Mixed lymphocyte reaction (MLR): the immunostimulatory capacity of *in vitro* generated UCB and PBL-DCs was assessed by MLR. Allogenic T cells were obtained from peripheral blood of healthy donors and were distributed at 10^5^ cells per well into round-bottomed 96-well micro plates (NUNC). Cells were co-cultured in the presence of graded numbers of irradiated DCs (2,500 rad, Co 60 source, BRIT, Navi Mumbai, India) in 200 μL of medium containing 10 % pooled AB^+^ plasma. Thymidine incorporation was measured on day 3 followingan 18-h pulse with [^3^H] thymidine (1 μCi/well) (BRIT, India) using standard procedures [25, 26, 31].

#### Generation of CTLs

CTLs were generated from UCB and PBL samples by our method as described earlier [30]. Briefly HLA-A*0201-positive UCB MNCs and PBL MNCs were used to generate DCs. Immature DCs were pulsed with MCF-7 lysate to a final concentration of 100 μg/ml of protein along with KLH (50 μg/ml) for 48 h as maturation stimuli in the culture medium for cross presentation of tumor antigen to autologous/allogenic sorted naïve T cells. For the negative control set of DCs, the maturation stimuli comprise 100 ng/ml each of LPS, CD40L and TNF-α, respectively. The autologous/allogenic naïve T cells were obtained by sorting of CD3-, CD8-, and CD45RA-positive cells using the FACS ARIA III (BD). The naïve T cells were co-cultured with MCF-7 lysate-pulsed DCs that were generated from the autologous/allogenic UCB or PBL sample. DC-T cell co-culture was maintained for 3 weeks with the addition of cytokines IL-2 (0.1 μg/ml) and IL-7 (5 μg/ml) and weekly re-stimulation with fresh antigen-pulsed DCs to generate effector cytotoxic killer T cells. Though obtaining HLA-A*0201-positive cord blood samples was relatively easier for CTL assay this was not true for peripheral blood samples. PBL were obtained as buffy coat samples from blood banks after getting institutional ethical clearance. Subsequently they were screened for HLA-A*0201 positivity, and only HLA A2-positive samples could be used for CTL assay. The frequency of obtaining HLA-A*0201 positivity was 1 in 10 samples screened. Procuring large volumes of HLA-A*0201-positive PBL samples (to isolate naïve T cells) also had ethical constraints. Moreover, literature suggests that a lower titration of target to killers have been used by many investigators [34, 35]. So we had taken into consideration the intermediate titration ratio of target to killers in this study (i.e., 1:0, 1:3……1:18). The experimental design for generation of CTLs is given in a flow chart in Additional file 2: Figure S2.

### Functional characterization of CTLs

CTLs were characterized as follows:

1. Assessment of activation by surface markers expression: on day 5 or day 7 of UCB/PBL, DC-T cell (DC-TC) co-culture cells were harvested and screened for the expression of early activation marker like CD69 along with CD25. After staining with CD69 and CD25 mAbs or with appropriate isotype controls, cells were acquired using the FACS Canto II from BD.
2. Intra cytoplasmic staining for granzyme A and B: CTLs generated *in vitro* were tested for the presence of these serine proteases enzymes within the intracellular compartments. Cells were harvested on day 21 of UCB/PBL DC-TC co-cultures and were stimulated by phorbol 12-myristate 13-acetate (PMA) (40 ng/ml) and ionomycin (100 ng/ml), along with Golgi stop Brefeldin A (1:1,000 diluted) for 4 h. After harvesting, the cells were subjected to surface staining with CD8 for 30 minutes. After fixation and permeabilization, granzyme A and B mAbs and appropriate isotype controls were added and the cells were incubated on ice for 60 minutes. Cells were acquired using the FACS Canto II from BD.
3. Streptamer staining for Mucin1 (MUC1)-STAPPVHNV-specific T cells: different specificities of CTLs arise when naïve T cells are cultured with pulsed DCs, depending on the antigen presentation capacity of the DCs. The cells were harvested on day 21 of UCB/PBL DC-TC co-culture. MUC1-STAPPVHNV [36] tagged with R-PE was used to estimate the presence of MUC1-specific CTLs as per the manufacturers’ instructions. After staining, cells were acquired using the FACS Canto II from BD. The schematic of the staining procedure is given in Additional file 3: Figure S3.
4. Cytokine profiling of DC-TC co-cultures by ELISA: supernatant from the *in vitro*-generated pulsed UCB-DCs or PBL-DCs and sorted naïve T cell co-cultures was collected on day 21 and stored at −20 °C. IL-12p70 and IL-10 or interferon gama (IFN-γ) content in the supernatants were assayed by ELISA using BD OptEIA™ ELISA kit (BD, Pharmingen) as previously reported [31]. The amount of secreted interleukins present in the samples was calculated by the standard curve method.

#### In vitro *CTL* assay

CTL assays were performed as described previously [31]. Briefly CTLs generated from unpulsed and pulsed sets of DCs were co-cultured with the target cells MCF-7 for 18 h in a flat bottom 96-well plate. Cells were then washed with plain media and then pulsed with [3H] thymidine for 8 h. Percent killing of MCF-7 was calculated by P-JAM assay using the formula:

$$ \%\ \mathrm{Killing} = \mathrm{CPMA}\ \left[\mathrm{Target}\ \mathrm{alone}\ \hbox{--}\ \mathrm{Target} + \mathrm{Killer}\right] \times 100/\mathrm{CPMA}\ \mathrm{Target}\ \mathrm{alone} $$

### *In vivo* CTL assay (xenograft model)

The NOD/LtSZ-scid/scid mice were purchased from Jackson Laboratories and were bred in the animal facility of our institute. The study was conducted adhering to the institution guidelines for animal husbandry and has been approved by the Institutional animal ethical committee-NCCS /Committee for the Purpose of Control and Supervision of Experiments on Animals (IAEC-NCCS/CPCSEA). Animal procedures involving intravenous (IV) infusion were carried out under anesthesia. The right flanks of age-matched female NOD/SCID mice, 4–6 weeks old, were subcutaneously injected with 4 × 10^6^ of MCF-7-luc-F5 cells in a total volume of 200 μL, which included 100 μL cell suspension in HBSS buffer plus 100 μL reduced growth factor Matrigel (Becton-Dickinson). Estradiol valerate USP (Cadila Healthcare Ltd.) (200 μg) was injected intraperitoneally (i.p.) per day from day zero until the tumor size reached a minimum of around 5 mm in lengthand in width, i.e., 62.5 mm^3^ in volume. One week after stopping estradiol injections, the mice were divided into three groups namely the control, the UCB-CTL and the PBL-CTL groups. The first day of imaging was considered as day zero. Before imaging the mice, tumor volume measurements were done for each mouse in all groups using digital Vernier calipers and the volume was calculated using the formula (length × width × width)/2. Mice were anesthetized by ketamine and xylazine (4:1 ratio) 0.05 ml per 20 grams of body weight. Then Luciferin D potassium salt (15 mg/ml) 0.2 ml was administered i.p. per mouse, followed by imaging using the IVIS 200 (Xenogen, PerkinElmer, MA, USA) and analysis by Living Image® (version 4.4). On the following day, each mouse in the UCB and PBL test group was infused via the tail vein with 10^7^ CTLs, generated from pulsed UCB and PBL-DCs, respectively. No CTLs were infused in the control sets. On the tenth day post CTL infusion, tumor volume measurements were taken and documented, followed by IVIS imaging as described above. On the same day, mice were sacrificed and the tumors were harvested and divided in two equal halves. One half was fixed in 10 % formalin for paraffin sectioning, followed by H&E staining. Images were taken using the Leica DMI6000 inverted microscope. The other half of the tissue was embedded in CRYO-OCT compound and kept frozen at −80 °C till further use. Shandon Cryotome was used to obtain 10-μ cryosections of frozen tissues. Mouse anti-human CD3 primary antibody and goat anti-mouse secondary antibody conjugated with Alexa Fluor® 488 was used in immunofluorescence staining to confirm the presence of infiltrating T cells in the xenograft tissue. Nuclei were stained with 4',6-diamidino-2-phenylindole (DAPI) to demarcate cellular structures and images were taken using the Zeiss LSM 510 Meta confocal microscope.

### Statistical analysis

Different variables of UCB-DCs/CTLs and PBL-DCs/ CTLs were compared. All results were expressed as means ± SD. Statistical analysis and graphs were prepared using SigmaPlot software (Version 11) (Jandel Scientific, CA, USA) and GraphPad Prism 6 Software (San Diego, CA, USA). All the statistical analysis of experiments comparing the two groups was evaluated by one-way analysis of variance (ANOVA). Probability values ≤0.05(*), ≤0.01(**) and ≤0.001(***) were considered statistically significant.

## Results

### UCB and PBL-derived DCs have similar morphology and phenotype

**Fig. 1:**
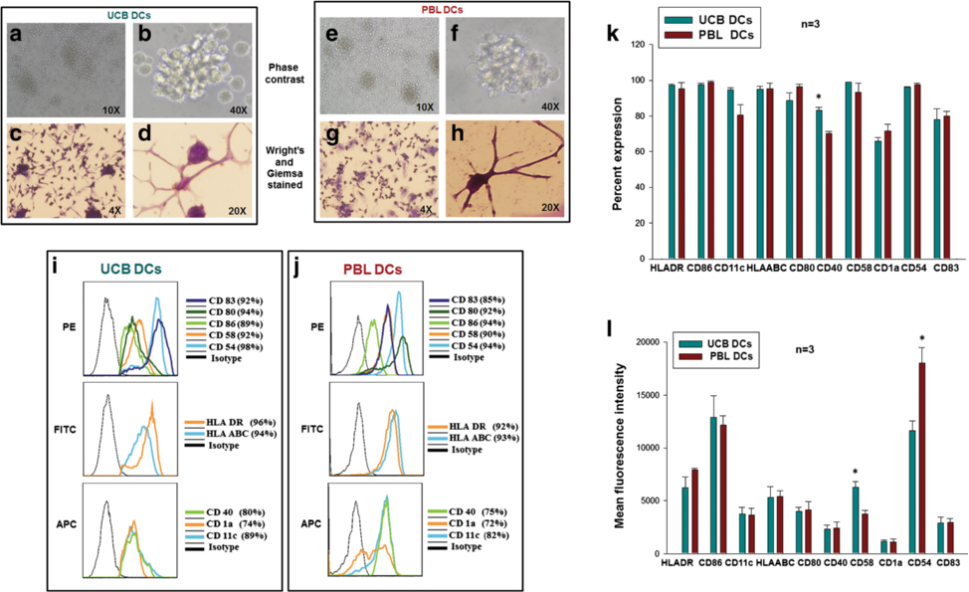
Morphology and phenotype of dendritic cells (DCs) generated from the two sources: Phase contrast images of umbilical cord blood DCs (*UCB*-*DCs*) (**a**, **b**) and peripheral blood PBL DCs (*PBL*-*DCs*) (**e**, **f**) at lower and higher magnification, respectively. Both sources generated the typical clusters of mature DCs. Wright-Giemsa-stained adherent cell clusters of UCB-DCs (**c**) and PBL-DCs (**g**) at lower magnification and morphology of single cells at higher magnification (**d**, **h**). FACS histogram overlay profile of a representative experiment from UCB-DCs (**i**) and PBL-DCs (**j**). *Black line* isotype control and *colored lines* specific CD molecules. Percent expression and mean fluorescence intensity (MFI), respectively, from three samples are shown in (**k**, **l**). It is evident that fully mature DCs were generated and there was no significant difference in the expression level of different surface markers, except for CD40, which was higher in UCB-DCs (**k**). In terms of MFI, CD58 was found significantly higher in UCB-DCs and CD54 in PBL-DCs (**l**). The data shown are mean ± SD, n = 3 (*p <0.05). *PE* phycoerythrin, *FITC* fluorescein isothiocyanate

DCs were generated from PBL monocytes and UCB MNCs as described earlier (a flow chart illustration is depicted in Additional file 1: Figure S1). The morphology of CD11c^+^ DCs from the two sources was studied by observing the culture plates under a phase contrast microscope (Fig. 1a, b, e, f) and by observing Wright-Giemsa stained culture plates (Fig. 1c, d, g, h). They showed stellate processes with a typical veiled appearance, dense cytoplasm, irregular nuclei and some cells in clumps, forming clusters. Wright-Giemsa stained cells showed long cytoplasmic projections i.e., dendrites, the hallmark of these cells. The DCs were further identified phenotypically by staining them with a panel of ten antibodies and acquiring on a flow cytometer. Representative FACS histogram profiles for UCB and PBL are depicted in Fig. 1i and j, respectively. Cumulative data for three samples (mean ± SD, n = 3) for percent CD expression is shown in Fig. 1k and MFI values in Fig. 1l. DCs generated from both sources had higher expression of DC-specific markers such as CD 1a, 11c, 80, 83, 86, etc. Typical DC-specific markers along with the co-stimulatory molecules, associated integrins and adhesion molecules were also expressed on the cells from both sources and were comparable. There was no significant difference in the percent expression of markers on DCs from the two sources, except for CD40 (Fig.1k), which was higher in UCB-DCs. There was also a significant difference in the MFI of CD58 (Fig. 1l), which was higher in UCB-DCs, and CD54 (Fig. 1l), which was higher in in PBL-DCs (*p* ≤0.05). These data underscore the fact that UCB-DCs are equivalent in nature to PBL DCs with respect to morphology and phenotype.

### UCB-DCs and PBL-DCs are functionally equivalent

**Fig. 2:**
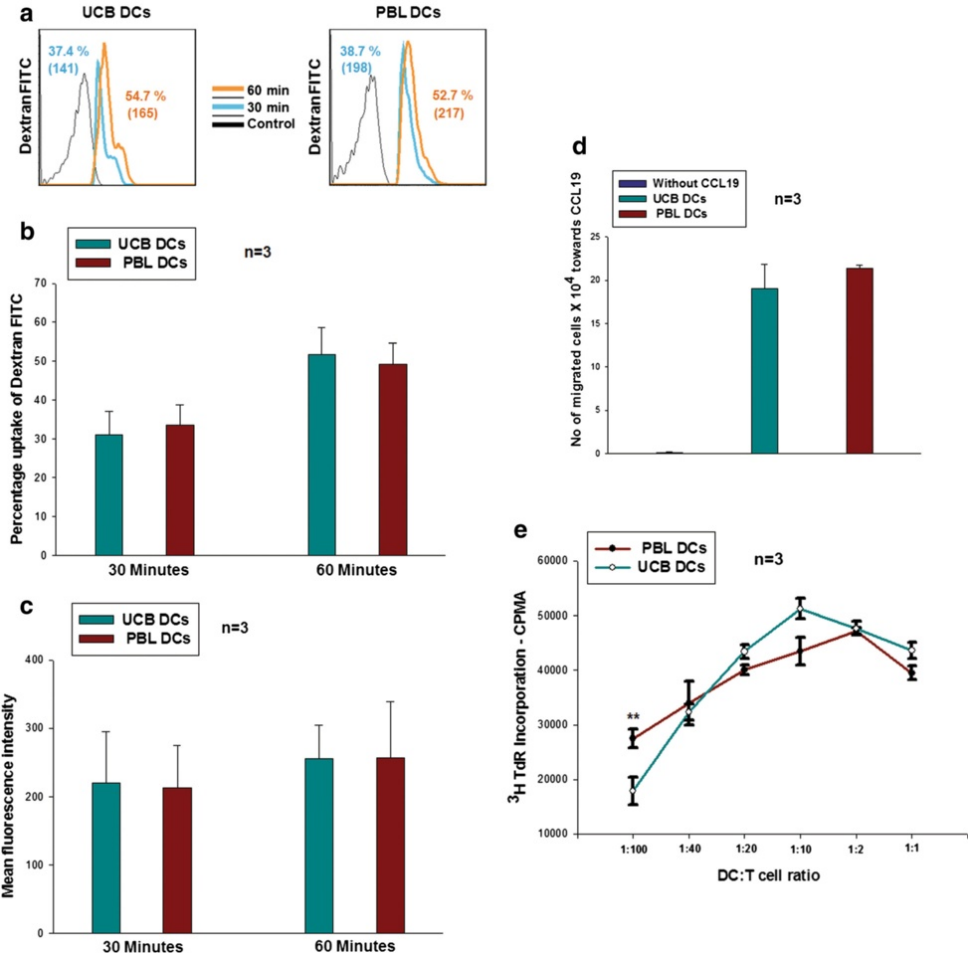
Functional characterization of dendritic cells (*DCs*). **a** FACS histogram overlays of dextran-fluorescein isothiocyanate (*FITC*) uptake profile of a representative experiment from umbilical cord blood DCs (*UCB*-*DCs*) and peripheral blood DCs (*PBL*-*DCs*) with mean fluorescence intensity (MFI) values in brackets, and **b** data from three experiments for percentage antigen uptake. **c** Cumulative MFI values from three samples. **d** Chemotaxis of DCs towards CCL-19. Low spontaneous migration was observed in the wells, without CCL-19, whereas both UCB-DCs and PBL-DCs efficiently migrated towards CCL-19 gradient. **e** Mixed lymphocyte reaction (MLR) from three independent experiments reveals that DCs generated from both sources exhibited similar MLR at a different effector-to-target ratio. The data shown are mean ± SD, n = 3 (**p <0.01). *CPMA* count per minute for beta

DCs have the characteristic property of activating naïve T cells, and this functionality depends upon the efficacy of their antigen uptake, chemotactic migration, antigen processing and presentation. We tested these functional attributes by performing *in vitro* assays, such as uptake of FITC-tagged dextran, chemotactic migration towards CCL-19, and MLR. We observed that both immature DC sets performed uptake of dextran-FITC within 30 and 60 minutes of the incubations tested. After 60 minutes of incubation time, there was 51 % uptake in UCB-DCs, whereas there was 49 % uptake in PBL-DCs. Figure 2a shows a histogram overlay profile of a representative sample with MFI values in brackets. Figure 2b shows cumulative data from three samples for percentage uptake of FITC-tagged dextran and Fig. 2c bar graph shows the cumulative MFI values from three samples (mean ± SD, n = 3). Migratory ability of the DCs towards CCL-19 was studied by *in vitro* assay using cell culture inserts as described earlier. As is evident from Fig. 2d (mean ± SD, n=3), migration ability was also similar between DCs from both sources. The MLR assay was performed by co-culturing DCs and T cells in different ratios and then checking the stimulatory capacity of DCs on T cells in terms of thymidine uptake. Figure 2e (mean ± SD, n = 3), clearly shows that both types of DCs elicited similar responses. Out of the six ratios tested, only at one ratio i.e., 1:100, did PBL-DCs appear to be significantly superior (*P* = 0.007); *P* values for the other ratios tested were 0.752, 0.107, 0.218, 0.775 and 0.258, respectively, which were not significant. This finding is important because MLR measures early events in the sensitization phase of antigen-specific cell-mediated immune response. With respect to these three attributes, we can conclude that UCB-DCs are as robust in functionality as PBL monocyte-derived DCs.

### Characterization of CTLs obtained from UCB and PBL-DCs

CTLs were generated by co-culturing sorted naïve HLA-A*0201-positive CD8+ T cells with HLA-A*0201-positive DCs, pulsed with MCF-7 lysate (a flow chart illustration is depicted in Additional file 3: Figure S3). CTLs thus generated by pulsed DCs from both sources were characterized for activation of surface markers such as CD69 and CD25, and intracellular levels of granzyme A and granzyme B. MUC1-specific CTLs were quantified by staining with MUC1-specific streptamer. Levels of secreted IFN-γ, IL-12p70 and IL-10 in the DCs and T cells co-cultured supernatant were assessed by ELISA.

#### *The expression of activation markers is significantly higher in UCB*-*derived CTLs*

**Fig. 3:**
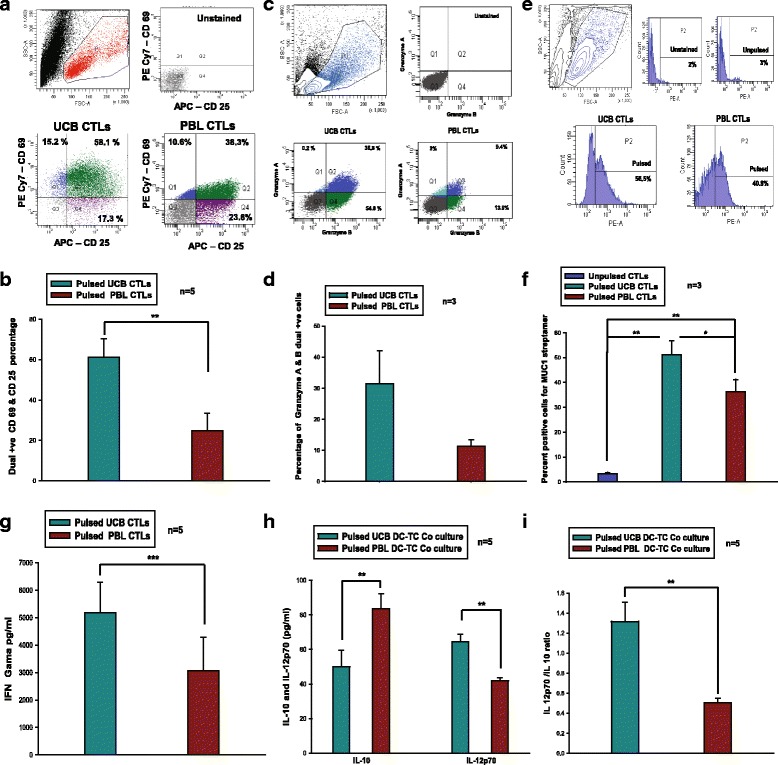
Functional characterization of CTLs: (**a**) Dot plot depicting the gating strategy and expression of activation markers CD69 & CD25 for a representative experiment from UCB/PBL. The dual positive cells from pulsed UCB/PBL-DCs are seen in upper right quadrant of lower panel. (**b**) Showing significantly higher CD69 & CD25 dual positive cells in UCB-derived CTLs than that of PBL-derived CTLs. (**c**) The gating strategy for intracellular staining of granzyme A & B in CTLs. Upper right quadrant in lower panel shows dual positive cells. (**d**) Showing presence of dual positive cells for granzyme A & B in pulsed UCB/PBL derived CTLs. (**e**) Dot plot and histogram showing the presence of MUC1 STAPPVHNV-specific T cells (lower panel) in total pool of CTLs from both the sources. (**f**) Showing significantly higher percentage of MUC1 specific TCRs in pulsed PBL/UCB-CTLs set as compared to unpulsed set. MUC1 specific TCRs in pulsed UCB-CTLs set was significantly higher than the PBL counterpart. (**g**) UCB-CTLs were capable of secreting significantly higher levels of IFN-γ than that of PBL-CTLs (n=5) in the co-culture derived from pulsed UCB/PBL-DCs. (**h**) Represents the levels of IL-10 and IL-12p70 in the co-culture. The UCB-DCs secreted significantly higher level of IL-12p70 and a significantly lower level of IL-10 than PBL-DCs. (**I**) IL-12 p70/IL-10 ratio in the co-culture. Ratio was significantly higher in cultures with UCB-DCs as compared to PBL DCs. The data shown are mean ± SD, n = 3/5 (*p < 0.05, **p < 0.01, ***p < 0.001)

Activation of T lymphocytes, both *in vivo* and *in vitro*, induces the expression of CD69. Induction of CD25 (the high affinity IL-2 receptor) and CD69 (the early activation antigen) is a characteristic feature of activated CTLs. Activation markers were detected on the cell surface using flow cytometry. Our data clearly show that CTLs generated from both sources express these two molecules. The FACS profile of one representative sample is shown in Fig. 3a. Figure 3b shows cumulative data from five samples (mean ± SD, n = 5). The data show that the CTLs from UCB-DCs appeared significantly more potent (*P* = 0.008) than their PBL counterparts in expression of these activation-associated markers.

#### Percentage of granzyme-positive cells is also higher in UCB-CTLs

A major pathway by which the CTLs induce their killing activity is via targeted exocytosis of cytoplasmic granules containing serine proteases, such as granzymes. Other locations where the granzymes can be detected are rough endoplasmic reticulum, golgi complex, and the trans-golgi reticulum, hence intracytoplasmic staining is needed to detect the presence of these proteases in the cells along with the use of Golgi stop (Brefeldin A). We found that the MCF-7 lysate-pulsed PBL-DC-CTLs/UCB-DC-CTLs produced granzyme A and B. The FACS profile is depicted in Fig. 3c. Cumulative data from three samples (mean ± SD, n = 3), showed a higher amount of these proteases in pulsed UCB-CTLs than in pulsed PBL-CTLs (Fig. 3d).

#### UCB-CTLs contain higher number of MUC1-specific CTLs

Out of all of the T cells in a given population, only a few may be specific for a given peptide against which its TCR match and are expanded on antigen presentation along with co-stimulation by APCs. To test the capability of MCF-7 lysate-pulsed UCB- and PBL-derived DCs to generate antigen-specific T cells after a secondary stimulation, sorted naïve HLA-A*0201-positive CD8^+^ T cells were weekly re-stimulated with freshly MCF-7 lysate pulsed HLA-A*0201-positive DCs. MUC1 is overexpressed in MCF-7 cells and it is also HLA-A*0201-restricted. The presence of MUC1-specific CD8^+^ T cells were quantitated by peptide-specific streptamer. Percentage of MUC1 peptide-STAPPVHNV-specific TCR present in the proliferating clones of T cells is represented in Fig. 3e and f, and in schematics in Additional file 3: Figure S3. We found a significantly higher percentage of streptamer-positive UCB-CTLs compared to PBL-CTLs. Figure 3e shows the FACS profile of one representative sample and Fig. 3f depicts cumulative data from three samples (mean ± SD, n = 3). These findings suggest that pulsed UCB-DCs and PBL-DCs were efficient in generating MUC1-specific CTLs.

#### UCB-CTLs secrete higher levels of IFN-γ

IFN-γ regulates multiple aspects of CD8^+^ T cell homeostasis as it promotes CD8^+^ T cell expansion and memory cells formation. CTLs are characterized by the secretion of IFN-γ. DCs from both sources were able to generate CTLs, which were capable of secreting a substantial amount of IFN-γ. However, pulsed UCB-CTLs were capable of secreting significantly larger amounts of IFN-γ, as compared to pulsed PBL-CTLs. Figure 3g represents cumulative data from five samples (mean ± SD, n = 5). It is evident from these data that UCB-CTLs are better than the PBL-CTLs in this respect.

#### Ratio of IL-12p70 to IL-10 was higher in supernatants of UCB-derived DC-T cell co-cultures

IL-12 is essential for ontogenesis of effector functions in T cells and in the establishment of functional memory. *In vitro* generated pulsed DCs were able to secrete higher levels of IL-12p70, as compared to IL-10. The UCB-DCs showed a better IL-12p70 secretion profile than PBL-DCs. Figure 3h represents the levels of IL-10 and IL-12p70 in the co-culture. The levels of IL-12p70 (pg/ml) were 64.41 ±4.31 and 41.84 ±1.73 for UCB and PBL-DCs, respectively. On the other hand, the level of IL-10 secretion by PBL-DCs was higher than for UCB-DCs. The levels of IL-10 (pg/ml) were 49.86 ± 9.72 and 83.53 ± 8.72 for UCB and PBL-DCs, respectively. Figure 3i represents the ratio of IL-12p70/IL-10. This ratio was significantly higher in UCB-DCs (mean ± SD, n = 5). It is evident from these data that the UCB-DCs have a more favorable T helper (Th)1 cytokine profile, as compared to PBL-DCs.

### The activated CD8^+^ T cells from both the DCs sources show comparable CTL activity

#### In vitro *CTL assay*

**Fig. 4:**
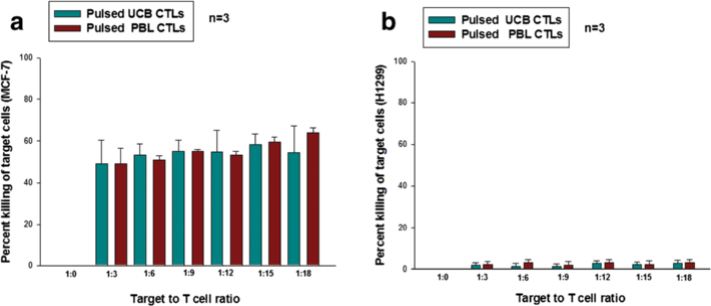
*In vitro* cytotoxic T lymphocyte (*CTL*) assay. **a** CTL assay data from three experiments depicting the killing efficacy of the target MCF-7 cells by CTLs generated by pulsed umbilical cord blood (*UCB*)/periperal blood (*PBL*)-dendritic cells (DCs). **b** Percent killing in the irrelevant target cell line H1229. Negligible killing effect was seen in these sets by pulsed UCB/PBL-DCs. Data shown are mean ± SD, n = 3

Figure 4a represents the cumulative data from three different biological replicates at different target-to-T-cell ratios. All the intermediate titration ratios of target-to-killers used in this study have shown that the DCs from the two sources have a similar killing effect. There was no spontaneous death as is evident for the 1:0 ratio where we saw zero percent killing as calculated by the formula in the P-JAM assay. This is depicted in Fig. 4a (x-axis 1:0 ratio). No significant difference was observed in the killing efficiency of CTLs derived from the two sources. As a negative control, CTLs were raised with un-pulsed UCB and PBL-DC sets. The CTLs thus generated were not effective in killing the target cells. To check the target specificity, CTLs obtained from MCF-7 lysate-pulsed UCB DCs were used in the CTL assay against H1299 and SH-SY5Y cell lines; a negligible killing effect was seen in these sets. Figure 4b shows the percent killing of the irrelevant target cell line H1229. The CPMA value in the SH-SY5Y control was 1,710 (SD ± 30), and in the pulsed and un-pulsed group at highest target-to-T-cell ratios were 1680 (SD ± 47) and 1,650 (SD ± 36), respectively. Additional file 4: Figure S4A and B depict the propinquity interaction of pulsed DCs and naïve T cells in the co-culture system. It is clearly seen (Additional file 4: Figure S4C) that CTLs from un-pulsed DCs do not cling to the target MCF-7 cells in the CTL assay. Additional file 4: Figure S4D and Additional file 4: Figure S4E illustrate the assailing nature of CTLs from pulsed DCs towards the target cells.

#### *In vivo CTL assay*-*xenograft model*

**Fig. 5:**
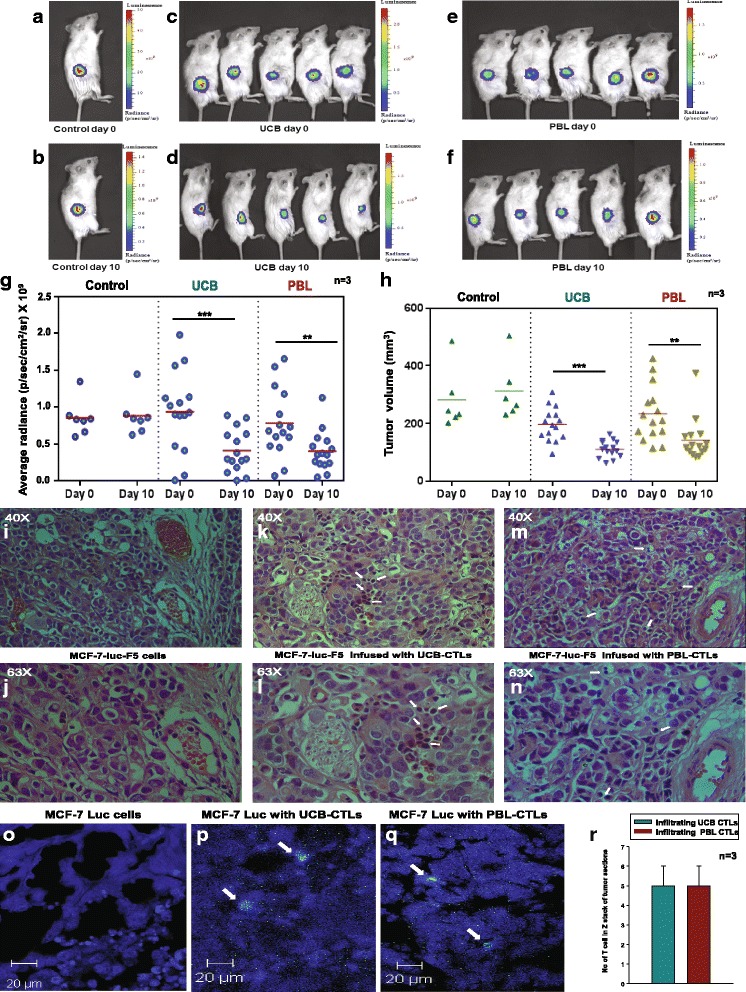
*In vivo* cytotoxic T lymphocyte (*CTL*) assay xenograft model. Representative image from one of the experiments showing average radiance (measured on the in vivo imaging system (IVIS)) from female NOD/SCID mice bearing tumor consisting of MCF-7-luc-F5 cells. **a**, **b** Average radiance from a control mouse on day 0 and day 10. **c** and **d**, **e** and **f** Average radiance from the umbilical cord blood (*UCB*)/peripheral blood (*PBL*)-CTLs group of mice on day 0 and day 10 post CTLs infusion. **g** Average radiance data of three experiments on day 0 and day 10 post CTLs infusion. There was significant regression in the tumor in both treatment groups infused with pulsed UCB/PBL-CTLs. *Horizontal lines* mean values ± SD. **h** Tumor volume on day 0 and day 10 post CTLs infusion. Similar significant reduction in the tumor mass was observed in both treatment groups infused with pulsed UCB/PBL-CTLs. **i**, **j** H&E-stained section of MCF-7-luc-F5 cell xenograft tumor from a control mouse showing no infiltrating T cells. **k** and **l**, **m** and **n** H&E-stained sections from a mouse infused with pulsed UCB/PBL-CTLs. Infiltrating CTLs in both treatment groups are observed (*arrows*). **o**-**q** Immunofluorescence images of sections stained with anti-human CD3 Ab, which further confirms the absence and presence of CTLs in the control and treatment group infused with pulsed UCB/PBL-CTLs, respectively. **r** Quantification of tumor-infiltrating T cells, which was enumerated by Z stack using the Zeiss confocal microscope. Data shown are mean ± SD, n = 3 (*P <0.05, ** *P* <0.01, *** *P* <0.001)

To assess the *in vivo* potency of the CTLs generated from both the sources, a xenograft model was used. Representative IVIS images of one experiment including control (not infused with CTLs), UCB and PBL groups on day 0 and day 10 are depicted in Fig. 5a and b, c and d, and e and f, respectively. There was marginal change in tumors in the control group, whereas in both the treated groups there was a significant decrease in the radiance. Three independent experiments having two or three mice in the control group and five mice in each test group were performed using 37 female NOD/SCID mice, 7 in the control set and 15 mice each in the UCB and PBL sets. Figure 5g represents cumulative data for average radiance (p/sec/cm^2^/sr) of three experiments. There was a significant regression in the tumor mass in the UCB group and PBL group on day 10 as compared to day 0, after CTL infusion. Figure 5h depicts the cumulative data for tumor volume (mm^3^) from three experiments, which shows that there was a significant decrease in tumor volume in the UCB group and the PBL group in the experimental time window, as compared to the control group. H&E staining for the MCF-7-luc-F5 xenograft tumor section for the control set is depicted in Fig. 5i and j. Figure 5k and l, and Fig. 5m and n represent UCB and PBL-CTLs infused test samples, respectively. CTL infiltrations were clearly evident (indicated by arrows). Figure 5o-q shows the immunofluorescence staining data for one slice of the section, which further confirms the T cell infiltration inside the tumor. Figure 5r bar graph shows the quantification of tumor-infiltrating T cells, which was enumerated by Z stack using the Zeiss LSM 510 Meta confocal microscope.

## Discussion

DC-based vaccination is an attractive immunotherapeutic strategy, because of its ability to induce tumor-specific T cell responses, which offers the desired anti-tumor effects with minimal toxicity. Allogenic DCs generated from healthy donors (HLA matching or partially matching) have been used in clinical trials as DC vaccines. These DCs initiated a CTL response and induced regression of the tumor [1]. Allogeneic DCs can also be generated from CD34^+^ cells derived from UCB. Our earlier studies had shown that UCB can serve as an alternative source for generating a homogenous DC population with high cell numbers [25, 26, 31]. However, as UCB are naïve cells, there is concern as to whether the DCs generated from them would elicit a CTL response as potent as that generated by PBL-derived DCs, and whether they could serve as good candidates for vaccines. To gain a better insight into this, we performed a systematic comparative study of standard PBL monocyte-derived DCs and UCB-generated DCs, with emphasis on CTL characterization and *in vitro* and *in vivo* CTL assays.

DCs generated from the two sources were similar in morphology and phenotype. Starting with 10^7^ MNCs per sample, 3.11 ± 1.495 × 10^7^ DCs were generated from three UCB samples and 0.53 ± 2.771 × 10^7^ DCs were generated from three PBL samples. Their functional attributes, such as antigen uptake capacity and ability to migrate towards a chemokine gradient of CCL-19 and MLR activity, i.e., potent immunostimulatory capacity, were equivalent. The CTLs generated were examined for activation markers (CD69 and CD25), granzyme A and B and quantitation of MUC1 peptide (STAPPVHNV)-specific CTLs by streptamer. Expression of granzyme A and B in human cytotoxic lymphocyte subsets as analyzed by flow cytometry have been reported by Grossman et al. [37]. The cytokines secreted by UCB-DCs and PBL-DCs in the co-culture system were favorable for a Th1 response. A low level of interleukins in the long term cultures is in accordance with the findings of Wong KL et al. [38]. Our data indicate that CTLs from UCB-DCs are superior to CTLs from PBL-DCs. Cytotoxic T lymphocytes are important constituents of an adaptive immune system. We tested the level of the known HLA-A*0201-restricted MCF-7-associated antigen, MUC1- STAPPVHNV peptide, by the streptamer assay and found that the pool of CTLs had a high percentage of MUC1-specific CTLs. As UCB T cells have different activation thresholds as compared to T cells in adults, it appears that the combination of UCB-DCs with UCB T cells generated superior levels of MUC1-specific CTL than that of PBL-DCs. However cross-stimulation of UCB-DCs with adult T cells, and vice-versa, would be required to ascertain if the increased potency was due to the presenting and/or responding cell type. Thus, a pure antigen-specific CD8^+^ T cell population could be generated, which could eventually be sorted out to give a single antigen-specific response in CTL assays. Our data are in agreement with those of Fernandez et al. [39] who described an *in vitro* system for the generation of functional, antigen-specific T cells from human stem cells, which could eventually provide a readily available cell source for adoptive transfer immunotherapies, and also enable better understanding of human T cell development.

Defense against virally-infected and malignant cells depends on the action of cytotoxic T lymphocytes and natural killer cells. Although these use several mechanisms to eliminate target cells, the principal event is secretion of cytotoxic granules. These granules contain the pore-forming protein perforin, together with a variety of granule-associated proteases, which include the granzymes. Target cell recognition by cytotoxic lymphocytes induces secretion of the cytotoxic granular content towards the target and the induction of death [40]. We saw high levels of granzyme A and B in UCB-DC CTLs, further highlighting their potential killing ability. The *in vitro* CTL activity was assessed by determining CTL killing efficiency with MCF-7 as target cells, and we found that CTLs from both sources exhibited equivalent killing efficiency. All the intermediate titration ratios of target-to-killers used in this study have shown that the DCs from the two sources have a similar killing effect. It appears that a higher titration ratio may also lead us to the same conclusions; however, we need to examine this aspect. Nevertheless, we feel that using a higher effector-target ratio may not change the scenario further in terms of interpretation of the current data. We also found evidence of cytotoxic activity specific to tumor antigens in the *in vitro* CTL assay, and not against nonspecific targets like H1229 and SH-SY5Y. These data clearly suggest that UCB-DCs/CTLs are as potent as PBL-DCs/CTLs. In contrast, they showed ameliorating features. Chang et al. [32] have also compared PBL-DCs with UCB-DCs using *in vitro* assays and they report similar findings. Signaling pathways play a decisive role in the differentiation, survival, expression of co-stimulatory molecules and antigen presentation capacity of DCs. They have shown that CBSC-derived DCs have quicker and greater extracellular signal-regulated kinase (ERK) and Akt phosphorylation, and weaker p38 phosphorylation, than peripheral blood mononuclear cell (PBMC)-derived DCs, when stimulated with LPS. In our study we have placed emphasis on the characterization of CTLs obtained from DCs of UCB /PBL and also focused on *in vivo* CTL assays using a xenograft in NOD/SCID mice.

Cord blood has many advantages over other sources, such as ready availability, low risk of severe Graft-versus-host disease and presence of stem cells with high proliferative potential. Thus, although UCB-DCs had greater capability in terms of vaccination and activation, there is a possibility of adverse effects associated with this line of therapy. These will have to be evaluated in pilot phases of clinical trials, thereby providing a better assessment and insight before moving forward with the other phases of clinical trials. Importantly, to compare the cancer vaccination or the stability of the two DCs from different resources, one must establish a competitive assay including UCB-DCs and PBL-DCs, employing immunosufficient mice. This experiment may yield valuable insight for our current understanding of effector functions in *in vivo* settings, which may help to verify the vaccination efficacy for the two DC sources. In other words, the future of UCB-derived DC vaccination depends largely on its *in vivo* behavior in patients, for which more stringent experiments on appropriate animal models need to be executed. Cord blood stem cells have longer telomeres and have longer survival. Monocytes from PBL are terminally differentiated cells, and therefore cannot proliferate. The major disadvantage of cord blood is the low number of stem cells but this is taken care of by the expansion step in our method. Cord blood banks from all over the world can be utilized for this purpose. When a stored sample is revived for transplantation, a small aliquot can be removed to generate DCs, which could then be transplanted into the patient, where they would migrate to lymph nodes and could elicit a strong T cell response against any residual tumor cells. de Haar et al. have already started such pre-clinical trials in the therapy of pediatric Acute myeloid leukemia using UCB-DCs [29]. Hutten et al. describe the first pre-clinical evidence for the suitability of UCB-DC either for the induction or for the reactivation of minor histocompatibility antigen (MiHA) HA-1-specific cytotoxic T cells. Their findings are of clinical significance in transplanted patients suffering from hematological malignancies [30].

To the best of our knowledge, we provide here for the first time, direct evidence in a xenograft model of a solid tumor, an adoptive T cell therapy by using ex vivo expanded CTLs having the antitumor activity derived from HLA-A*0201-positive UCB and PBL samples. We found that UCB/CTLs and PBL/CTLs had immunotherapeutic effects against MCF-7-luc-F5 solid tumors in female NOD/SCID mice. It is evident from the IVIS data that there is a significant remission in tumors 10 days after CTL infusion. The H&E and immunofluorescence staining of tumor sections revealed substantial homing and infiltration of CD8^+^ T cells. It needs to be determined whether frequent booster doses of CTLs over longer period could achieve complete regression. It is also important to generate DCs by our method from other sources such as CD34^+^ from mobilized PBL or enriched apheresis samples and test their killing efficacy, using a different cell line in *in vivo* xenograft experimental models. We have come a long way since the first clinical trial with autologous pulsed DCs to stimulate anti-tumor immunity in humans. We are still in the quest to find the answer to the main problem i.e., what is required to evoke a therapeutic immunity against cancer, which emerges by evading the defense system of the host. With the advancement in technology our perspective on the role of DCs as adjuvant in cancer immunotherapy has broadened remarkably. We are at the point of entering a new era, in which immunotherapy is going to revolutionize the treatment of almost every kind of cancer.

## Conclusions

Our data conclusively show that UCB-derived DCs are equally potent as the standard source of DC vaccines, i.e., PBL monocyte-derived DCs. In other words, UCB-DCs or the CTLs derived from them could be used effectively in allogeneic anti-tumor vaccines for immunotherapy, as an alternative to peripheral blood or bone marrow-derived DCs/CTLs.

## Additional files

Additional file 1: Figure S1. Flow chart depicting experiment design for generation of dendritic cells (*DCs*). *UCB* umbilical cord blood, *MNC* mononuclear cells, *GM*-*CSF* granulocyte-macrophage colony stimulating factor, *PBL* peripheral blood, *LPS* lipopolysaccharide. (TIFF 2506 kb) Additional file 2: Figure S2. Flow chart depicting experiment design for generation of cytotoxic T lymphocytes (CTL). *UCB* umbilical cord blood, *PBL* peripheral blood, *LPS* lipopolysaccharide, *DC* dendritic cells. (TIFF 3354 kb) Additional file 3: Figure S3. Schematics for Streptamer staining. *CTLs* cytotoxic T lymphocytes. (TIFF 2289 kb) Additional file 4: Figure S4. Depicts the interaction of pulsed dendritic cells (DCs) and naïve T cells. **A** Clonal expansion of naïve T cells during co-culture with pulsed DCs. **B** Larger magnification to show the tethering of many naïve T cells with a mature DC. **C** Negligible interaction of unpulsed cytotoxic T lymphocytes (*CTLs*) with the target MCF-7 cells. **D**, **E** Flocking of CTLs derived from pulsed peripheral blood (PBL)/umbilical cord blood (*UCB*) DCs to the target MCF-7 cells. (TIFF 2950 kb)

## Abbreviations

APC: antigen presenting cells
BSA: bovine serum albumin
CCL-19: chemokine (C-C motif) ligand 19
CD40L: CD40 ligand
CPMA: count per minute for beta
CTL: ctotoxic T lymphocyte
DC: dendritic cell
DMEM: Dulbecco’s modified Eagle’s medium
DMSO: dimethyl sulfoxide
ELISA: enzyme-liniked immunosorbent assay
FITC: fluorescein isothiocyanate
FLT3-L: fms-like tyrosine kinase-3 ligand
GM-CSF: granulocyte-macrophage colony stimulating factor
H&E: hematoxylin and eosin
HSC: hematopoietic stem cell
IFN-γ: interferon gama
IL: interleukin
IVIS: in vivo imaging system
kDa: kiloDaltons
KLH: keyhole limpet hemocyanin
LPS: lipopolysaccharides
mAb: monoclonal antibody
MFI: mean fluorescence intensity
MHC: major histocompatibility complex
MLR: mixed lymphocyte reaction
MNC: mononuclear cell
MUC1: Mucin 1
NCCS: National Centre for Cell Science
PBL: peripheral blood
PBS: phosphate-buffered saline
PE: phycoerythrin
PMA: phorbol 12-myristate 13-acetate
SCF: stem cell factor
TCR: T cell receptor
Th: T helper
TNF-α: tumor necrosis factor-alpha
TPO: thrombopoietin
UCB: umbilical cord blood
