# Supplementary figures and images for: Umbilical cord blood-derived CD11c+ dendritic cells could serve as an alternative allogeneic source of dendritic cells for cancer immunotherapy
Source: Stem Cell Res Ther. 2015 Sep 25;6:184. doi: 10.1186/s13287-015-0160-8 (PMC4583174; doi:10.1186/s13287-015-0160-8)

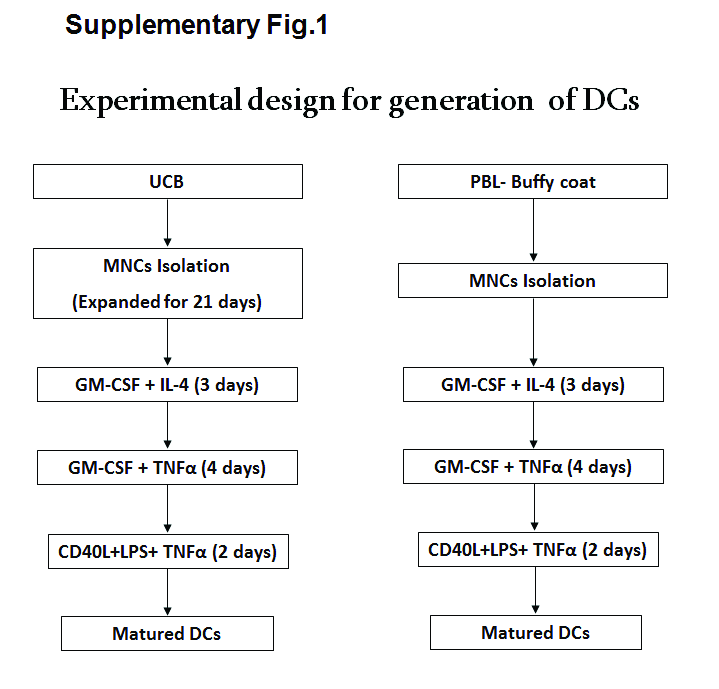

Supplement: Additional file 1: Figure S1. — Flow chart depicting experiment design for generation of dendritic cells (DCs). UCB umbilical cord blood, MNC mononuclear cells, GM-CSF granulocyte-macrophage colony stimulating factor, PBL peripheral blood, LPS lipopolysaccharide. (TIFF 2506 kb) [file 13287_2015_160_MOESM1_ESM.tif]

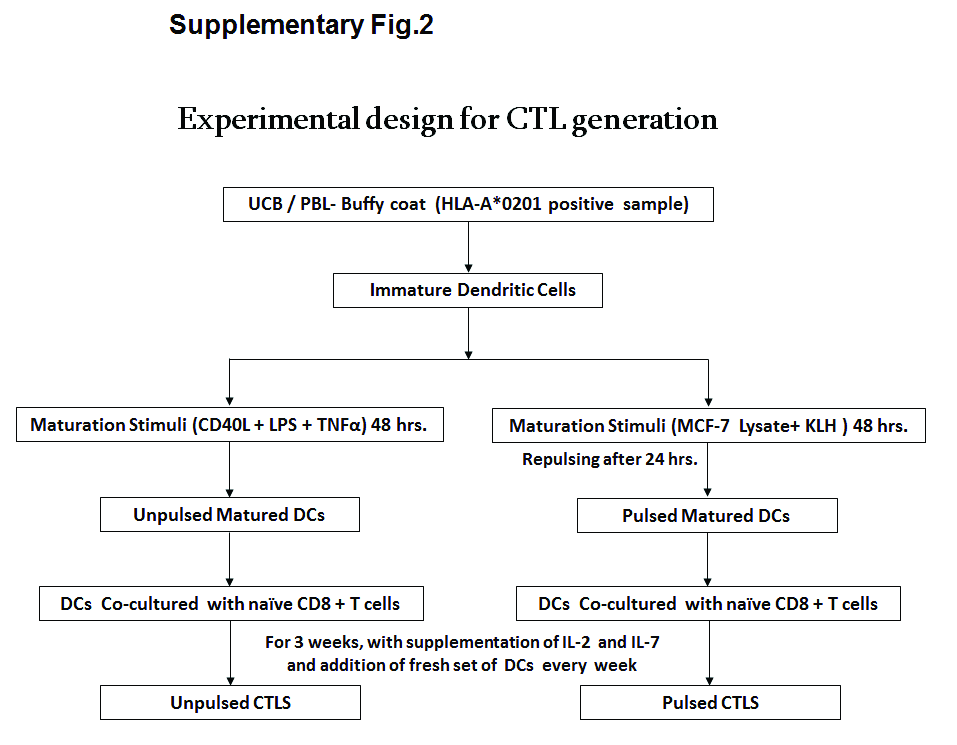

Supplement: Additional file 2: Figure S2. — Flow chart depicting experiment design for generation of cytotoxic T lymphocytes (CTL). UCB umbilical cord blood, PBL peripheral blood, LPS lipopolysaccharide, DC dendritic cells. (TIFF 3354 kb) [file 13287_2015_160_MOESM2_ESM.tif]

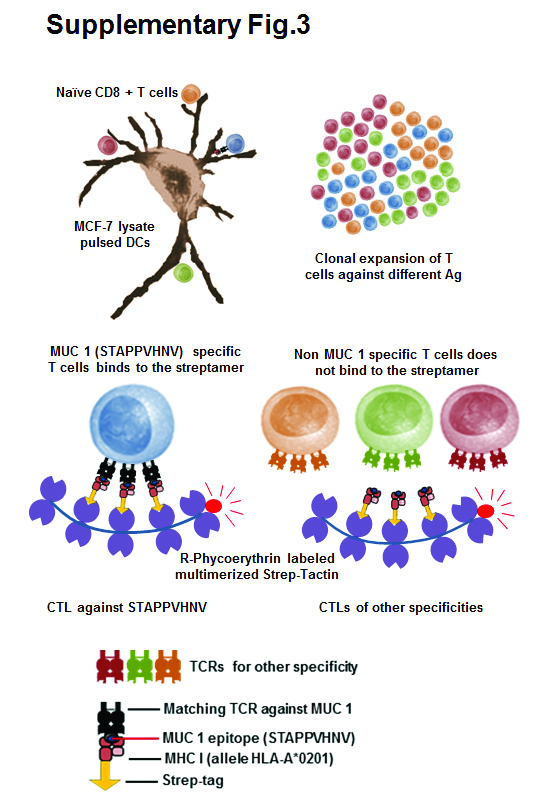

Supplement: Additional file 3: Figure S3. — Schematics for Streptamer staining. CTLs cytotoxic T lymphocytes. (TIFF 2289 kb) [file 13287_2015_160_MOESM3_ESM.tif]

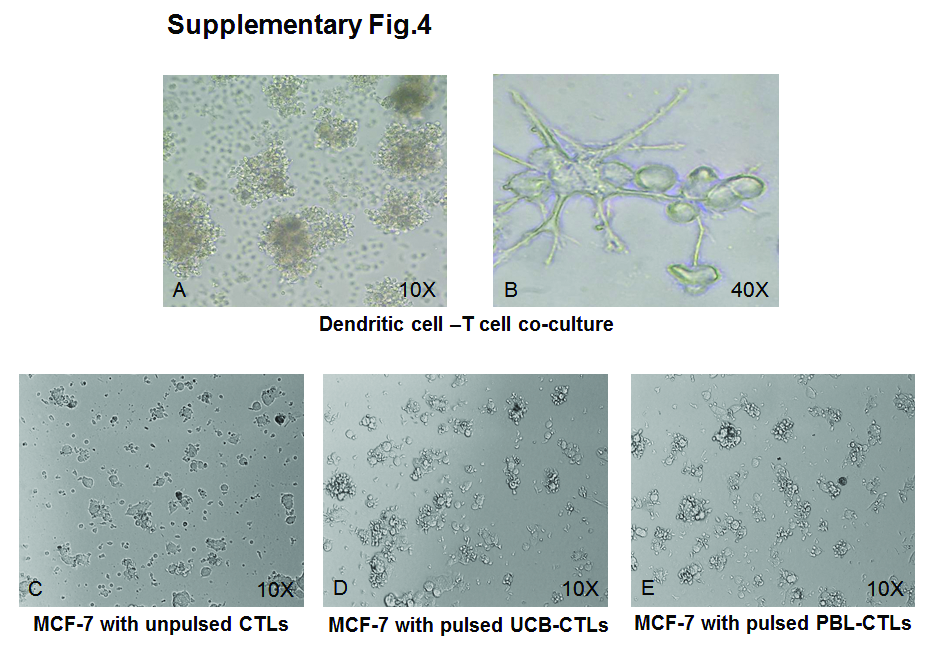

Supplement: Additional file 4: Figure S4. — Depicts the interaction of pulsed dendritic cells (DCs) and naïve T cells. A Clonal expansion of naïve T cells during co-culture with pulsed DCs. B Larger magnification to show the tethering of many naïve T cells with a mature DC. C Negligible interaction of unpulsed cytotoxic T lymphocytes (CTLs) with the target MCF-7 cells. D, E Flocking of CTLs derived from pulsed peripheral blood (PBL)/umbilical cord blood (UCB) DCs to the target MCF-7 cells. (TIFF 2950 kb) [file 13287_2015_160_MOESM4_ESM.tif]
